# The Impact of Wheat Bran on the Morphology and Physiology of the Gastrointestinal Tract in Broiler Chickens

**DOI:** 10.3390/ani10101831

**Published:** 2020-10-08

**Authors:** Qinghui Shang, Di Wu, Hansuo Liu, Shad Mahfuz, Xiangshu Piao

**Affiliations:** State Key Laboratory of Animal Nutrition, College of Animal Science and Technology, China Agricultural University, Beijing 100193, China; sqh123hxj456@163.com (Q.S.); superwudee@163.com (D.W.); liuhansuo1115@163.com (H.L.); shadmahfuz@yahoo.com (S.M.)

**Keywords:** antioxidant status, broilers, digestive enzyme activities, gastrointestinal development, morphology, nutrient digestibility

## Abstract

**Simple Summary:**

Recently, dietary fiber has gained special attention due to its various beneficial effects on poultry. In poultry, moderate amounts of insoluble dietary fiber have been shown to be beneficial to nutrient utilization by improving the physiology of the gastrointestinal tract. Therefore, this study used wheat bran as a source of insoluble fiber to investigate wheat bran on digestive function in broiler chickens. The results indicate that supplementation of 30 g/kg wheat bran enhanced nutrient digestibility by improving antioxidant status, gizzard development, intestinal digestive enzyme activities and morphology in broilers. In conclusion, wheat bran could be used for improving feed efficiency in broilers.

**Abstract:**

There is increasing evidence showing that moderate amounts of insoluble dietary fiber can improve nutrient utilization by positively influencing the physiology of the gastrointestinal tract. The present study was conducted to investigate the effects of wheat bran as a source of insoluble fiber on nutrient digestibility, serum antioxidant status, gastrointestinal development, digestive enzyme activities and intestinal morphology in broiler chickens. A total of 96 one-day-old male Arbor Acre broiler chickens were assigned to two treatments with six replicate cages per treatment and eight birds per replicate for 42 d. Dietary treatments consisted of the control group (CON, control diet) and wheat bran group (WB, 30 g/kg wheat bran). Inclusion of WB increased (*p* < 0.05) the digestibility of dry matter, organic matter, gross energy and crude protein on Day 42. Birds fed WB had lower (*p* < 0.05) serum total cholesterol concentration on Day 21, and lower (*p* < 0.05) serum concentrations of low-density lipoprotein, total cholesterol and total triglyceride on Day 42. Inclusion of WB increased (*p* < 0.05) serum glutathione peroxidase activity on Day 21 and superoxide dismutase activity on Day 42, but tended (*p* = 0.07) to decrease serum malondialdehyde concentration on Day 21, and significantly decreased (*p* < 0.05) serum malondialdehyde concentration on Day 42. Birds fed WB had a greater (*p* < 0.05) relative weight of gizzard on both Day 21 and 42. Inclusion of WB increased (*p* < 0.05) activities of amylase and trypsin in pancreas and jejunal mucosa on Day 21, and increased (*p* < 0.05) amylase activity in pancreas and jejunal mucosa. Birds fed WB had greater (*p* < 0.05) villus height and villus height to crypt depth ratio in jejunum and ileum on Day 42. In conclusion, supplementation of 30 g/kg WB enhanced nutrient digestibility by improving antioxidant status, gizzard development, intestinal digestive enzyme activities and morphology of broilers.

## 1. Introduction

At present, developing new feeding strategies for healthier digestive system development and more efficient nutrient utilization of broilers has received increasing attention [1,2]. Traditionally, dietary fiber (DF) has been considered a diluent of the diet or even an anti-nutritional factor [3]. However, DF has attracted increasing attention due to various beneficial effects on gut health [4]. However, further studies demonstrated that there is a lack of consistency with regard to the effects of DF, suggesting that the effects of DF depend on their physicochemical characteristics [5,6]. Soluble dietary fiber (SDF) generally has a viscous structure, thus increasing digesta viscosity and reduces passage rate, and eventually reduces nutrient utilization [3]. In contrast, insoluble dietary fiber (IDF) has a nonviscous structural component, and recent studies in poultry have demonstrated that moderate amounts (20–30 g/kg) of IDF to be beneficial to nutrient utilization by improving gastrointestinal development and enzyme secretion [6,7]. Therefore, supplementing insoluble dietary fiber to broiler diets may be a feasible method to improve feed efficiency.

Wheat bran (WB), a byproduct of the milling process, is rich in insoluble fiber, consisting mainly arabinoxylans and, to a lesser extent, cellulose and β-glucans [8]. Previous studies have mostly focused on how to eliminate the anti-nutritional effects of a high level of WB [9]. However, with the development of research and exploration, the WB has been shown to be involved in the regulation of gastrointestinal physiology such as gastric emptying time and intestinal transit rate, which may consequently influence digestive function [10]. Besides, some studies demonstrated that WB plays an important role in metabolic regulation (particularly in lipid metabolism) in rats and humans, which is associated with the digestion and absorption of nutrients [11,12]. In addition, arabinoxylan or arabinoxylooligosaccharides derived from WB has been shown to improve gut health such as gut microbiota, which may in turn improve digestive traits [13,14]. Furthermore, in vitro experiments have proved that WB has antioxidant properties due to the presence of phenolic acids, which is favorable to the maintenance of gut homeostasis [15]. All these properties of WB suggest that moderate amounts of WB may have a beneficial impact on feed efficiency in broilers. However, there is limited evidence regarding the effects of moderate amounts WB on digestive function in broilers. Therefore, the present study was conducted to evaluate the effects of 30 g/kg WB on nutrient digestibility in broiler chickens. Serum biochemical parameters, antioxidant status, gastrointestinal development, digestive enzyme activities and intestinal morphology were also evaluated to explore the underlying mechanism.

## 2. Materials and Methods

The experimental procedures and use of animals in this study were approved by the Institutional Animal Care and Use Committee of China Agricultural University (Beijing, China; No. AW09089103-1).

### 2.1. Birds, Diets and Management

A total of 96 one-day-old male Arbor Acre broiler chickens were purchased from a local commercial hatchery and allocated to 2 dietary treatments in a randomized complete design; each treatment had 6 replicate cages of 8 birds per cage (0.9 × 0.6 × 0.4 m). Dietary treatments included the control group (CON, basal diet) and the wheat bran group (WB, 30 g/kg wheat bran). All diets were formulated to meet or exceed the nutrient requirements recommended by the National Research Council (NRC) (1994) [16] and fed in mash form (Table 1). Chromium oxide (Cr_2_O_3_, 0.25%) was added to all diets as an indigestible marker.

The study consisted of two periods, namely, starter (Day 1 to 21) and finisher (Day 22 to 42). Birds were housed in wire-floored cages in an environmentally controlled room and had free access to feed and water throughout the trial period. Light was on continuously. The room temperature was set at 33 °C for the first 3 d, and then decreased to 24 °C at a rate of 3 °C each week; thereafter, the temperature was maintained until the end of the experiment.

### 2.2. Sample Collection

From Day 40 to 42, excreta samples were collected twice a day from pans beneath each cage and stored at −20 °C. At the end of the collection period, excreta samples were thawed, pooled within cage, mixed thoroughly, and dried at 65 °C for 72 h.

On Day 21 and 42, 1 bird per cage, with the body weight (BW) close to the mean BW of that cage, was selected. Blood samples were collected by wing vein puncture and centrifuged at 3000× *g* for 15 min at 4 °C. Then, the serum was harvested and stored at −80 °C until analysis. After blood samples’ collection, birds were euthanized by electrical stunning and bleeding of the carotid artery. The gastrointestinal tract was carefully excised, and adherent mesentery and fat were removed. The empty weight of proventriculus, gizzard and small intestine were determined and expressed as relative weight (g/kg live BW). The length of small intestine was also recorded and expressed in relative length (cm/kg live BW). The pancreas was collected, snap frozen in liquid nitrogen, and stored at −80 °C until analysis. Sections from the middle parts of duodenum, jejunum and ileum (about 3 cm in length) were collected and fixed in 10% phosphate-buffered formalin for morphological evaluation. The remaining jejunum section was gently flushed with 0.9% ice-cold saline, and then the mucosa was scraped with a sterile glass slide, snap frozen in liquid nitrogen and stored at −80 °C.

### 2.3. Chemical Analysis

Ingredients, diets and excreta samples were ground through a 1-mm screen and then analyzed for dry matter (DM; AOAC, 2007; method 930.15), crude protein (CP; AOAC, 2007; method 976.05) and ash (AOAC, 2007; method 942.15) [17]. The neutral detergent fiber (NDF) and acid detergent fiber (ADF) were determined using fiber analyzer (Ankom Technology, Macedon, NY, USA) according to Van Soest, et al. [18]. The gross energy (GE) was determined using an automatic adiabatic oxygen bomb calorimeter (Parr 6300 Calorimeter, Moline, IL, USA). The total dietary fiber (TDF) and IDF were analyzed using AOAC (2007) [17] methods 985.29 and 991.43, respectively. The SDF was calculated as the difference between TDF and IDF. The chromium (Cr) content was measured using anatomic absorption spectrophotometer (Z-5000; Hitachi, Tokyo, Japan) according to the procedure of Williams et al. [19]. The apparent total tract digestibility (ATTD) values were calculated by the equation as follows: ATTD (%) = [1 − (Cr_diet_ × Nutrient_excreta_)/(Cr_excreta_ × Nutrient_diet_)] × 100%, where Cr_diet_ represents the chromium content in diet, and Cr_excreta_ represents the chromium content in excreta. Nutrient_excreta_ represents the nutrient content in excreta, and Nutrient_diet_ represents the nutrient content in diet.

### 2.4. Serum Biochemical Parameter Analysis

Serum concentrations of urea nitrogen (UN), high-density lipoprotein cholesterol (HDL-C), low-density lipoprotein cholesterol (LDL-C), total cholesterol (TC), triglyceride (TG), total protein (TP), albumin (ALB), and glucose (GLU) were measured by the use of commercial kits (Sino-UK Institute of Biological Technology, Beijing, China) using a fully automatic biochemical analyzer (Hitachi High-Technologies Corporation, Hitachi 7160, Japan).

### 2.5. Serum Antioxidant Status Analysis

The total-antioxidant capacity (T-AOC), malondialdehyde (MDA) concentration, and activities of superoxide dismutase (SOD) and glutathione peroxidase (GSH-Px) in serum were determined with commercially available kits (Nanjing Jiancheng Bioengineering Institute, Nanjing, China) according to the manufacturer’s instructions.

### 2.6. Digestive Enzyme Activity Analysis

The pancreas and jejunal mucosa were thawed at 4 °C and homogenized in 9 vols of 0.9% ice-cold saline in a tissue grinder. Homogenates were centrifuged at 15,000 × *g* for 15 min at 4 °C and the supernatant was collected for digestive enzyme assays. The activities of amylase, lipase and trypsin were measured by commercial kits (Jiancheng Bioengineering Institute, Nanjing, China) according to the manufacturer’s instructions. One unit of amylase activity was defined as the amount of amylase to hydrolyze 10 mg of starch in 30 min at 37 °C. One unit of lipase activity unit was defined as the amount of lipase to hydrolyze 1 μmol of triglyceride in 1 min at 37 °C. One unit of trypsin activity was defined as the amount of trypsin to hydrolyze 1 µmol of benzoyl-L-arginine ethyl ester in 1 min at 37 °C. To normalize the enzyme activity, total protein concentration in pancreas and jejunal mucosa was determined by a bicinchoninic acid protein assay kit (Jiancheng Bioengineering Institute, Nanjing, China). Enzyme activities were expressed as U/mg or U/g protein.

### 2.7. Intestinal Morphology Analysis

Intestinal samples were removed from 10% phosphate-buffered formalin, then dehydrated through a graded ethanol series (70 to 100%), cleared with xylene and embedded in paraffin wax. Serial sections (5 μm thickness) were cut with a LEICA RM2135 rotary microtome (Leica Microsystems GmbH, Wetzlar, Germany), and stained with hematoxylin and eosin. A minimum of 15 intact and well-oriented villi and their associated crypts from each segment were measured at 40× magnification with a light microscope (CK40, Olympus, Tokyo, Japan). Villus height was measured from the tip of the villi to the villus crypt junction, and crypt depth was defined as the depth of the invagination between adjacent villi.

### 2.8. Statistical Analysis

All data were analyzed by student’s t-test for unpaired data with cage as an experimental unit using SAS software (version 9.2; SAS Inst. Inc., Cary, NC). Significant difference was declared at *p* < 0.05, and tendency was declared at 0.05 ≤ *p* < 0.10.

## 3. Results

### 3.1. Nutrient Digestibility

The effects of WB supplementation on nutrient digestibility in broilers on Day 42 are presented in Table 2. Dietary inclusion of WB increased (*p* < 0.05) DM, OM, GE and CP digestibility compared with CON. Dietary treatments did not influence NDF and ADF digestibility.

### 3.2. Serum Biochemical Parameters

The effects of WB supplementation on serum biochemical parameters in broilers on Day 21 and 42 are presented in Table 3. On Day 21, there were no differences in serum concentrations of UN, HDL-C, LDL-C, TG, TP, ALB and GLU between treatments. However, birds fed WB had (*p* < 0.05) lower serum TC concentration than those fed CON. On Day 42, serum concentrations of HDL-C, TP, ALB and GLU still did not differ among treatments. However, compared with CON, dietary inclusion of WB decreased (*p* < 0.05) serum concentrations of LDL-C, TC and TG, and tended (*p* = 0.06) to decrease serum concentration of UN.

### 3.3. Serum Antioxidant Status

The effects of WB supplementation on serum biochemical parameters in broilers on Day 21 and 42 are presented in Table 4. On Day 21, serum SOD activity and T-AOC were not affected by dietary treatments. However, birds fed WB had greater (*p* < 0.05) serum GSH-Px activity and tended (*p* = 0.07) to have lower serum MDA concentration when compared with CON. On Day 42, none of the dietary treatments led to remarkable changes in serum GSH-Px activity and T-AOC. However, serum SOD activity was greater (*p* < 0.05) in WB than in CON. In addition, the lower serum MDA concentration was observed in birds fed WB compared with those fed CON (*p* < 0.05).

### 3.4. Gastrointestinal Development

The effects of WB supplementation on gastrointestinal development in broilers on Day 21 and 42 are presented in Table 5. The relative weight of proventriculus and small intestine, as well as the relative length of small intestine did not significantly vary with any of the dietary treatments on Day 21 or 42. However, birds fed WB had a greater (*p* < 0.05) relative weight of gizzard than those fed CON on both Day 21 and 42.

### 3.5. Digestive Enzyme Activities

The effects of WB supplementation on digestive enzyme activities in pancreas and jejunal mucosa of broilers on Day 21 and 42 are shown in Table 6. On Day 21, supplementation of WB increased (*p* < 0.05) activities of amylase and trypsin in pancreas and jejunal mucosa when compared with CON. No differences were found in lipase activity in pancreas and jejunal mucosa among treatments. On Day 42, birds fed WB also had greater (*p* < 0.05) amylase activity in pancreas and jejunal mucosa than those fed CON. Moreover, birds fed WB tended (*p* = 0.09) to have greater trypsin activity in pancreas than those fed CON. Dietary treatments did not affect the activities of lipase and trypsin in jejunal mucosa.

### 3.6. Intestinal Morphology

The effects of WB supplementation on intestinal morphology in broilers on Day 42 are shown in Table 7. In duodenum, there was no difference in the villus height, crypt depth, and villus height to crypt depth ratio among treatments. However, in jejunum, compared with CON, the WB increased (*p* < 0.05) villus height and villus height to crypt depth ratio. A similar pattern was also observed in ileum. The supplementation of WB increased (*p* < 0.05) villus height and villus height to crypt depth ratio when compared with CON.

## 4. Discussion

In the present study, supplementation of 30 g/kg WB significantly increased digestibility of DM, OM and GE, which was consistent with Jimenez-Moreno et al. [20], who reported that fiber inclusion (30 g/kg cellulose, oat hulls or sugar beet pulp) increased apparent total tract retention of DM, OM and nitrogen in broilers. The beneficial effects of WB on nutrient digestibility may be attributed to the effects of WB on gastrointestinal development and the concomitant increase in digestive enzyme secretion [3]. Similar results were also reported by Donadelli et al. [6], in which birds fed fiber containing diet (30 g/kg cellulose, beet pulp, or miscanthus grass) had greater ATTD of DM, OM and GE in broilers compared with those fed the no fiber dietary treatment. In contrast, the negative effects of DF on nutrient digestibility were also observed in previous studies [21,22]. The inconsistent results may be related to many factors such as fiber source, additional levels, and composition of the basal diet, with effects being more pronounced when moderate amounts of insoluble fiber are added to low-fiber basal diets [3,23].

Generally, serum biochemical parameters reflect the physiological and metabolic status in the body, among which the TC, TG, LDL-C, and HDL-C concentrations can reflect the lipid metabolism [24]. In the present study, it was found that the concentration of TC was significantly decreased in birds fed WB than those fed CON, which was consistent with Abdel-Hafeez et al. [25]. One plausible explanation for the decrease in TC concentration is that WB, as a source of insoluble fiber, may reduce transit time of digesta in the gastrointestinal tract, thereby increasing cholesterol excretion [26]. Another plausible explanation is that DF may bind some of the bile acids and phospholipids present in the digesta, thereby reducing emulsification and absorption of dietary lipids [27]. This study also found that inclusion of WB reduced serum LDL-C concentration compared with CON. Similarly, Heidarisafar et al. [28] also observed that feeding 5 or 10% apple peel waste (a fibrous ingredient) decreased serum LDL-C concentration in broilers. The lower LDL-C concentration observed in WB may be attributed to the possible mechanism of antioxidant and antiperoxide lowering activity of WB [25].

Antioxidant enzyme systems, such as SOD and GSH-Px, remove excessive free radicals and protect against oxidative damage, thereby maintaining the overall health of poultry [29]. The MDA is a major byproduct of lipid peroxidation, and its concentration in serum indirectly indicates the degree of cellular lipid peroxidation and the accumulation of reactive oxygen species [30]. In the present study, the lower serum concentration of MDA in birds fed WB suggested a lower degree of lipid peroxidation and the concomitant decrease in free radicals. Furthermore, an increase in serum SOD activity of birds fed WB indicated that WB may relieve oxidative stress by enhancing antioxidant enzymes activity. It has been well demonstrated that phenolic acids concentrated in WB are strong antioxidants and could alleviate oxidative stress by quenching or neutralizing reactive species [31], which may be a reason responsible for the improved serum antioxidant status in WB treatment. Therefore, the results confirmed that WB could improve antioxidant enzyme activities to remove excessive free radicals, eventually resulting in better antioxidation function.

The gizzard is the principal physical feed-processing organ in poultry [32]. Generally, a well-developed gizzard is associated with powerful contractions of the muscular layers, which not only ensures the complete grinding of the feed, but also increases intestinal refluxes, facilitating the mixing of the digesta with digestive enzymes [7]. It has been documented that diet composition is important to the development of the gizzard [33]. In this study, the increased relative weight of the gizzard in WB at both evaluating periods was a good indicator of improved functioning of the gizzard, which may contribute to improve nutrient digestibility. The results of this study were supported by the previous reports, which have shown an improvement in the size and weight of the gizzard in broilers when dietary fiber was included [34,35,36]. The fiber fraction in the diet is usually retained longer in the gizzard as they are hard to ground to a certain critical size that allows them to pass through the pyloric sphincter, which stimulates mechanically the development of the muscular layers of the gizzard [7,36]. Consequently, the gizzard was heavier in broilers fed WB than those fed other diets in the present study.

Digestive enzymes such as amylase, lipase and protease in the digestive tract are responsible for the hydrolysis of dietary nutrients, and their activities play a vital role in the digestive efficiency of nutrients [37]. In the current study, the activities of amylase and trypsin in the pancreas and jejunum were increased in the WB group, which was in agreement with the improved nutrient digestibility. Inconsistent with our findings, Yokhana et al. [37] reported that insoluble fiber supplementation increased proteolytic digestive enzyme activities in the proventriculus and pancreas of broilers. Kheravii et al. [38] demonstrated that dietary fiber upregulated the expression of genes encoding digestive enzymes in broilers. One possible reason for the increased enzyme activity was that WB could stimulate enzyme secretion by regulating the production of hormones, such as cholecystokinin, secretin and gastrin produced in the digestive tract [39]. Another possible reason was that WB supplementation could increase the population of some bacteria that can deliver enzymes, thus increasing the intestine digestive enzyme activity [40].

Intestinal morphological characteristics, including villus height, crypt depth and villus height to crypt depth ratio, are accepted as an indicator for intestinal health and are directly related to the absorptive capacity of the mucous membrane [41]. Intestinal villi are the site where most of the nutrients are absorbed, and their height is indicative of the absorptive capacity of intestinal mucosa [42]. Crypts are known as the villus factory where new epithelial cells are produced, and their depth reflects the rate of tissue turnover to ensure renewal of the villus [43]. The villus height to crypt depth ratio is a useful criterion for estimating the absorptive capacity of the small intestine [44]. In the present study, increased villus height of the jejunum and ileum with broilers fed WB suggests an increased surface area that would be capable of greater absorption of the available nutrients. Moreover, the WB supplementation increased the villus height to crypt depth ratio of the ileum, confirming the better intestinal absorptive capacity again. The results are probably related to the beneficial effects of DF on gut microbiota composition and associated metabolites [44]. Similarly, Rezaei et al. [45] observed the positive effects of insoluble fiber on the ileal villus height to crypt depth ratio. However, in contrast to our results, Jiménez-Moreno et al. [46] demonstrated that sugar beet pulp reduced villus height and crypt depth and tended to reduce villus height to crypt depth ratio in the jejunum. These discrepancies may be closely associated with addition level and the source of fiber used. In the study of Jiménez-Moreno et al. [46], 7.5% of sugar beet pulp was used and an excessive amount of fiber abraded the small intestine in the mucosal surface and shortened villus height [47]. As a source of soluble fiber, inclusion of sugar beet pulp could increase digesta viscosity, which may, in turn, elevate anaerobic bacterial activity and subsequently change the villus characteristics [40].

## 5. Conclusions

In conclusion, supplementation of 30 g/kg WB could enhance nutrient digestibility by improving antioxidant status, gizzard development, intestinal digestive enzyme activities and morphology of broilers.

## Figures and Tables

**Table 1:** Composition and nutrient levels of the experimental diets (g/kg, as-fed basis).

Item	Day 1–21		Day 22–42	
	CON ^1^	WB^2^	CON	WB
Corn	581.7	539.7	642.6	600.6
Soybean meal	304.4	304.4	240.5	240.5
Fish meal	20.0	20.0	20.0	20.0
Corn gluten meal	20.0	20.0	25.0	25.0
Wheat bran	-	30.0	-	30.0
Soybean oil	33.8	45.8	36.0	48.0
Dicalcium phosphate	15.0	15.0	10.4	10.4
Limestone	13.0	13.0	13.5	13.5
Salt	3.0	3.0	3.0	3.0
L-Lysine HCl, 78%	0.1	0.1	0.8	0.8
DL-Methionine, 98%	1.4	1.4	0.4	0.4
L-Threonine, 98%	0.1	0.1	0.3	0.3
Chromium oxide	2.5	2.5	2.5	2.5
Premix^3^	5.0	5.0	5.0	5.0
Calculated nutrient composition				
Metabolic energy, MJ/kg	12.77	12.77	13.19	13.19
Available Phosphorus	4.5	4.5	3.5	3.6
Analyzed nutrient composition				
Crude protein	210.8	210.4	190.0	190.8
Calcium	10.4	11.1	9.2	9.5
Total phosphorus	7.0	7.3	5.9	6.1
Lysine	12.0	11.7	11.0	11.1
Methionine	5.5	5.6	4.2	4.0
Threonine	8.2	8.6	7.6	7.9
Tryptophan	3.1	3.0	2.6	2.3
Total dietary fiber	139.4	147.2	134.7	142.4
Insoluble dietary fiber	121.3	128.7	117.0	124.3
Soluble dietary fiber	18.1	18.5	17.7	18.1

^1^CON: control diet; ^2^WB: 30 g/kg wheat bran diet; analyzed composition of wheat bran (as-fed basis) was 89.37% dry matter, 5.82% ash, 83.55% organic matter, 17.12% crude protein, 17.01 MJ/kg gross energy, 37.36% neutral detergent fiber, 11.55% acid detergent fiber, 44.57% total dietary fiber, 3.89% soluble dietary fiber and 40.68% insoluble dietary fiber. ^3^Premix supplied per kg diet: vitamin A, 11,000 IU; vitamin D, 3,025 IU; vitamin E, 22 mg; vitamin K_3_, 2.2 mg; thiamine, 1.65 mg; riboflavin, 6.6 mg; pyridoxine, 3.3 mg; cobalamin, 17.6 μg; nicotinic acid, 22 mg; pantothenic acid, 13.2 mg; folic acid, 0.33 mg; biotin, 88 μg; choline chloride, 500 mg; iron, 48 mg; zinc, 96.6 mg; manganese, 101.76 mg; copper, 10 mg; selenium, 0.05 mg; iodine, 0.96 mg; cobalt, 0.3 mg.

**Table 2:** Effects of wheat bran on nutrient digestibility in broilers on Day 42.

Item	CON	WB	SEM	*p*-Value
Dry matter	0.73	0.75	0.002	0.02
Organic matter	0.77	0.79	0.002	0.02
Crude protein	0.63	0.67	0.009	0.03
Gross energy	0.76	0.78	0.003	0.01
Neutral detergent fiber	0.45	0.46	0.021	0.84
Acid detergent fiber	0.20	0.18	0.008	0.26

CON, control diet; WB, 30 g/kg wheat bran diet; SEM, standard error of mean.

**Table 3:** Effects of wheat bran on serum biochemical parameters in broilers on Day 21 and 42.

Item	CON	WB	SEM	*p*-Value
Day 21				
Urea nitrogen, mmol/L	1.40	1.38	0.05	0.74
High-density lipoprotein mmol/L	2.36	2.07	0.10	0.14
Low-density lipoprotein, mmol/L	0.39	0.33	0.02	0.11
Total cholesterol, mmol/L	3.25	2.78	0.12	0.03
Triglyceride, mmol/L	0.56	0.43	0.08	0.37
Total protein, g/L	23.21	24.14	1.12	0.58
Albumin, g/L	11.53	11.60	0.44	0.91
Glucose, mmol/L	12.27	12.27	0.64	0.99
Day 42				
Urea nitrogen, mmol/L	1.22	1.16	0.02	0.06
High-density lipoprotein cholesterol, mmol/L	1.06	0.93	0.04	0.11
Low-density lipoprotein cholesterol, mmol/L	0.30	0.14	0.02	< 0.01
Total cholesterol, mmol/L	1.67	1.03	0.10	< 0.01
Triglyceride, mmol/L	0.35	0.28	0.02	0.03
Total protein, g/L	11.57	10.33	0.97	0.53
Albumin, g/L	6.22	4.92	0.38	0.17
Glucose, mmol/L	8.82	8.12	0.30	0.20

CON, control diet; WB, 30 g/kg wheat bran diet; SEM, standard error of mean.

**Table 4:** Effects of wheat bran on serum antioxidant status in broilers on Day 21 and 42.

Item	CON	WB	SEM	*p*-Value
Day 21				
Total antioxidant capacity, U/mL	1.14	1.59	0.24	0.26
Superoxide dismutase, U/mL	32.10	32.51	1.07	0.81
Glutathione peroxidase, µmol/L	31.55	36.33	0.67	0.01
Malondialdehyde, nmol/L	7.45	6.65	0.20	0.06
Day 42				
Total antioxidant capacity, U/mL	2.99	3.24	0.58	0.79
Superoxide dismutase, U/mL	38.62	47.69	1.59	< 0.01
Glutathione peroxidase, µmol/L	31.92	37.90	2.22	0.21
Malondialdehyde, nmol/L	7.08	4.87	0.52	0.02

CON, control diet; WB, 30 g/kg wheat bran; SEM, standard error of mean.

**Table 5:** Effects of wheat bran on gastrointestinal development in broilers on Day 21 and 42.

Item	CON	WB	SEM	*p*-Value
Day 21				
Relative weight of proventriculus, g/kg BW	6.53	7.04	0.25	0.18
Relative weight of gizzard, g/kg BW	19.76	24.78	0.70	< 0.01
Relative weight of small intestine, g/kg BW	34.95	39.17	1.46	0.06
Relative length of small intestine, cm/kg BW	189.39	202.37	9.41	0.38
Day 42				
Relative weight of proventriculus, g/kg BW	3.20	3.63	0.20	0.17
Relative weight of gizzard, g/kg BW	11.33	12.70	0.25	0.01
Relative weight of small intestine, g/kg BW	26.61	27.63	1.30	0.62
Relative length of small intestine, cm/kg BW	61.02	63.98	1.81	0.39

CON, control diet; WB, 30 g/kg wheat bran diet; SEM, standard error of mean; BW, body weight.

**Table 6:** Effects of wheat bran on digestive enzyme activities in pancreas and jejunum of broilers on Day 21 and 42.

Item	CON	WB	SEM	*p*-Value
Day 21				
Pancreas				
Amylase, U/g	185.09	227.03	10.94	0.02
Lipase, U/mg	43.30	47.94	2.29	0.21
Trypsin, U/mg	20.73	26.52	1.35	0.04
Jejunum				
Amylase, U/g	166.22	209.91	7.51	<0.01
Lipase, U/mg	13.40	15.43	1.24	0.28
Trypsin, U/mg	4.40	5.63	0.30	0.02
Day 42				
Pancreas				
Amylase, U/g	234.37	275.29	8.91	0.01
Lipase, U/mg	56.38	65.16	3.79	0.16
Trypsin, U/mg	24.63	33.02	3.06	0.09
Jejunum				
Amylase, U/g	190.63	233.16	11.30	0.02
Lipase, U/mg	15.60	16.96	0.54	0.11
Trypsin, U/mg	6.32	7.41	0.50	0.16

CON, control diet; WB, 30 g/kg wheat bran diet; SEM, standard error of mean.

**Table 7:** Effects of wheat bran on intestinal morphology in broilers on Day 42.

Item	CON	WB	SEM	*p*-Value
Duodenum				
Villus height, µm	2080	2242	65.88	0.12
Crypt depth, µm	263	263	17.34	0.97
Villus height to crypt depth ratio	8.17	8.65	0.61	0.60
Jejunum				
Villus height, µm	1333	1610	50.17	0.01
Crypt depth, µm	248	236	16.47	0.60
Villus height to crypt depth ratio	5.50	6.95	0.41	0.03
Ileum				
Villus height, µm	1121	1388	62.32	0.01
Crypt depth, µm	165	164	12.41	0.92
Villus height to crypt depth ratio	6.86	8.64	0.40	0.01

CON, control diet; WB, 30 g/kg wheat bran diet; SEM, standard error of mean.
